# Differentiation of the Chinese minority medicinal plant genus *Berchemia* spp. by evaluating three candidate barcodes

**DOI:** 10.1186/s40064-016-2207-4

**Published:** 2016-06-03

**Authors:** Li-Cheng Guo, Ming-Ming Zhao, Wei Sun, Hong-Li Teng, Bi-Sheng Huang, Xiang-Pei Zhao

**Affiliations:** Hubei University of Chinese Medicine, Wuhan, 430065 China; Guangxi Institute of Minority Medicine, Nanning, 530001 China; Institute of Chinese Materia Medica, China Academy of Chinese Medical Sciences, Beijing, 100700 China; Medical Laboratory College, Beihua University, Jilin, 132013 China

**Keywords:** Chinese minority medicinal plants, *Berchimia* spp., ITS2 DNA barcode, Species identification, Pairwise distance analysis

## Abstract

The genus *Berchemia* comprises important Chinese plants with considerable medicinal value; however, these plants are often misidentified in the herbal medicinal market. To differentiate the various morphotypes of *Berchemia* species, a proficient method employing the screening of universal DNA barcodes was used in this work. Three candidate barcoding loci, namely, *psbA*-*trnH*, *rbcL*, and the second internal transcribed spacer (ITS2), were used to identify an effective DNA barcode that can differentiate the various *Berchemia* species. Additionally, PCR amplification, efficient sequencing, intra- and inter-specific divergences, and DNA barcoding gaps were employed to assess the ability of each barcode to identify these diverse *Berchemia* plants authentically; the species were differentiated using the Kimura two-parameter and maximum composite likelihood methods. Sequence data analysis showed that the ITS2 region was the most suitable candidate barcode and exhibited the highest interspecific divergence among the three DNA-barcoding sequences. A clear differentiation was observed at the species level, in which a maximum distance of 0.264 was exhibited between dissimilar species. Clustal analysis also demonstrated that ITS2 clearly differentiated the test species in a more effective manner than that with the two other barcodes at both the hybrid and variety levels. Results indicate that DNA barcoding is ideal for species-level identification of *Berchemia* and provides a foundation for further identification at the molecular level of other Rhamnaceae medicinal plants.

## Background

*Berchemia* is a genus of plants in the Rhamnaceae family, which comprises 32 deciduous woody plants located in Asia, South America, and Africa (Huxley and Griffiths 1999). In China, *Berchemia* consists of 19 native species (Chen and Dong 2006), which are primarily distributed in the south, southwestern, and eastern regions (Sinicae 1988). These species include climbing plants or small- to medium-sized trees, several of which are endangered but offer significant medicinal values; these important plants include *B. lineatai* (Shen et al. 2010) and *B. berchemiafolia* (Kitamura and Murata 1984; Fu and Jin 1992; Ohwi 1984). In Japan, the roots, stems, and leaves of *Berchemia* plants are used to treat liver diseases, neuralgia, and gall stones; furthermore, these parts are utilized in traditional Chinese medicine (Mukhtar et al. 2004).

The characteristics, transection structure, and powder properties of *Berchemia* species demonstrate obvious distinct features that can be used for microscopic identification. In particular, *B. lineata*, *B. polyphylla*, and *B*. *polyphylla* var. leioclada are closely related in terms of microstructure and microscopic characteristics. However, these three species can be distinguished on the basis of the characteristics of their leaf edge; the leaf edge cells of *B*. *lineata* are round, and the cell walls are not thickened, or thickening is not obvious. The leaf edge cells of *B. polyphylla* are square and rectangle, and the cell walls are obviously thickened. The leaf edge cells of *B. polyphylla* var. leioclada are round, and the cell walls show obvious thickening (Teng et al. 2010; Ye et al. 2013). These distinctions can provide a basis for the pharmacognostical identification of *Berchemia* species.

*Berchemia* species are highly similar in terms of apparent vegetative morphology and thus often misidentified. In Chinese herb markets, different species under the same name are sold as dried roots. Distinguishing these species merely by sight is impossible to the untrained eye. Although all the *Berchemia* species provide medicinal value, consuming a wrong one reduces drug efficiency and causes ill effects after prolonged usage. Therefore, the development of an accurate method to prove the authenticity of plant raw materials is necessary because traditional methods, including organoleptic trait evaluation and phytochemical and pharmacognostic methods, cannot accurately identify species (Yan et al. 2013).

DNA barcoding is a rapidly developing frontier technology that is gaining worldwide attention. This novel technology uses a standardized genomic DNA sequence from a standard locus as a species identification tool (Kress et al. 2005) and has become popular in species identification (Gregory 2005; Miller 2007). Barcoding is a convenient tool to identify species for nonprofessional users, such as traditional drug producers, forensic specialists, and customs officers (Xue and Li 2011). Numerous DNA barcodes exist in plants and animals, which can be used to identify species. CO1, which is used as a DNA barcode, is a powerful tool for the discrimination of closely related species in most animals (Hebert et al. 2003). In 2009, the Plant Working Group of the Consortium for the Barcode of Life (CBOL) recommended that the loci *rbcL* + *matK* can be used as core barcodes to identify plants (CBOL Plant Working Group, 2009). The *psbA*-*trnH* intergenic spacer and internal transcribed spacer (ITS)/ITS2 were also suggested as barcodes for plant identification at the Third International Barcode Conference in Mexico City (Chen et al. 2010; Kress et al. 2005). Yao et al. (2010) proposed that the ITS2 locus, a popular phylogenetic marker, should be used as a universal DNA barcode and a complementary locus for CO1 to identify plants and animals, respectively (Yao et al. 2010). Pang et al. (2012) suggested that the *tmH*-*psbA* + ITS2 combination performs better or equally well in taxonomic groups, as compared with other combinations, such as *matK* + *rbcL* (Pang et al. 2012).

The present work aimed to distinguish different *Berchemia* species by screening three candidate loci, namely, *rbcL*, *psbA*-*trnH*, and ITS2, as the core barcodes and by identifying the most suitable barcode to accurately identify the members of the *Berchemia* genus. Furthermore, this study aimed to provide drug safety references for current medical fields.

## Results and discussion

### Amplification and sequence analysis

Genomic DNA was extracted from 55 samples belonging to seven species of *Berchemia*. The regions ITS2, *psbA*-*trnH*, and *rbcL* underwent effective amplification for all the selected samples. All PCR products corresponding to these three barcodes were successfully sequenced, and high-quality bidirectional sequences were obtained. The PCR amplification size for ITS2, *psbA*-*trnH*, and *rbcL* ranged within 491–561, 364–470, and 729–757 bp, respectively. Table 1 shows that the amplification efficiency of ITS2 and *rbcL* was 100 %, and that of *psbA*-*trnH* was 92 %. These results indicated that the three barcodes were applicable for the following analysis. ITS2 presented variable sites in 17/226 bp of the aligned sites, of which 11 were parsimony-informative, whereas *psbA*-*trnH* and *rbcL* showed very low variations of 6/430 bp and 5/551 bp, respectively (Table 2).

**Table 1:** Amplification efficiency of 50 *Berchemia* samples using five selected markers

	Samples	Quantity	ITS2	*psbA-trnH*	*rbcL*
	*B. floribund*	10	10	9	10
	*B. polyphylla*	10	10	9	10
	*B. sinica*	5	5	4	5
	*B. kulingensis*	5	5	5	5
	*B. polyphylla var.* leioclada	10	10	10	10
	*B. lineata*	10	10	9	10
	*B. hirtella*	5	5	5	5
Total		55	55	51	55
Amplification efficiency (%)			100	92	100

**Table 2:** Evaluation of the three DNA markers used in the present study

	*rbcL*	*psbA*-*trnH*	ITS2
PCR success (%)	100	100	100
Amplified product length (bp)	729–757	364–470	491–561
Aligned sequence length (bp)	551	430	226
No. of variable sites	5	6	13
No. of Pi sites	4	5	11
No. of singletons	1	0	2
Inter specific distance mean	0.001	0.002	0.026
Total no. of clusters (UPGMA)	3	3	4
Identification efficiency (%)	15	50	100

### Pairwise distance analysis

The mean interspecific genetic distances of the evaluated DNA regions are listed in Table 2. In the ITS2 region, the *Berchemia* interspecific distance mean was 0.026; however, the distance means of the two other candidate barcodes were 0.001 (*rbcL*) and 0.002 (*psbA*-*trnH*). The sequence data were further considered for pairwise distance analysis, and the ITS2 gene region was proven the most suitable for species differentiation (Table 2). In this study, the morphologically similar species *B. kulingensis* and *B*. *polyphylla* showed a distance of 0.009. The three other morphologically similar species, namely, *B*. *polyphylla*, *B. floribunda*, and *B*. *sinica*, showed a distance of 0.014. Furthermore, the morphologically similar varieties of *B*. *lineata* and *B*. *polyphylla* var. leioclada showed a distance of 0.000 (Table 3).

**Table 3:** Pairwise analysis of the ITS2 region using the Maximum Composite Likelihood method

	TB01	DH02	DY03	KL04	GE05	GZ06	GB07
TB01							
DH02	0.023						
DY03	0.018	0.014					
KL04	0.028	0.023	0.009				
GE05	0.023	0.018	0.014	0.023			
GZ06	0.000	0.028	0.023	0.032	0.018		
GB07	0.032	0.018	0.023	0.032	0.018	0.028	

### Clustal analysis

**Fig. 1:**
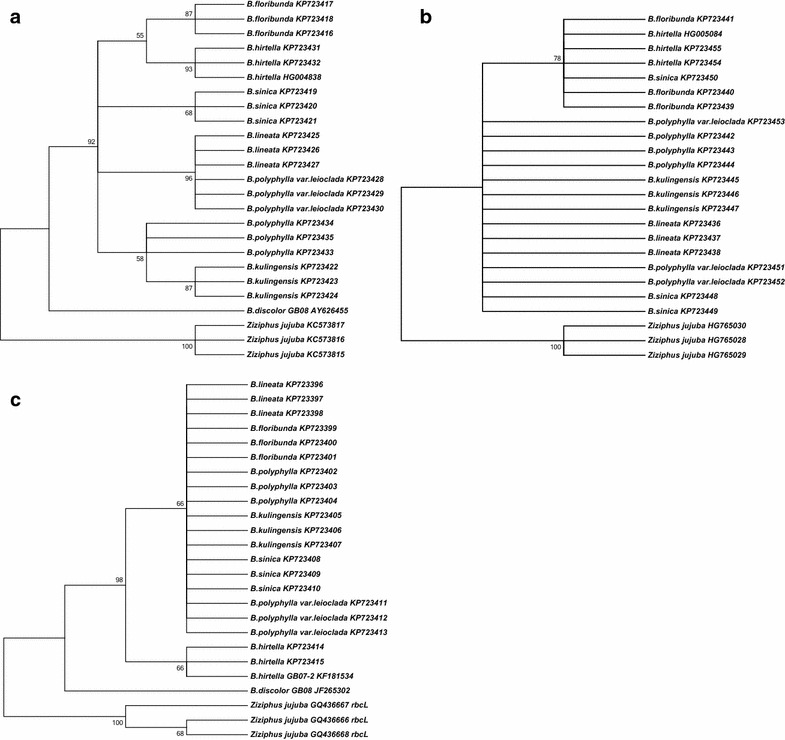
Evolutionary history inferred using the neighbor-joining method. **a** ITS2, **b** *psbA*-*trnH*, and **c** *rbcL*

In this study, 55 ITS2, 51 *psbA*-*trnH*, and 55 *rbcL* sequences were obtained from seven selected species. The five other sequences, two ITS2 sequences (*B*. *hirtella* HG004838; *B*. *discolor* AY626455), two *rbcL* sequences (*B. hirtella* KF181534; *B*. *discolor* JF265302), and a *psbA*-*trnH* sequence (*B. hirtella* HG005084) were downloaded from GenBank. To evaluate the feasibility of the three candidate barcodes to differentiate the species, Clustal analysis was conducted using the neighbor-joining (NJ) method, and *Ziziphus jujube* belonging to Rhamnaceae was employed as outgroup. Following the phylogenetic analysis, the ITS2 region was clearly differentiated among all eight species. Overall, 21 selected ITS2 sequences from seven species and an ITS2 sequence belonging to another species obtained from the NCBI database were aligned in the NJ tree. As shown in Fig. 1a, each of the same species was divided into one group at the species level. Only the subspecies *B*. *polyphylla* var. leioclada was clustered to *B. lineata*. As shown in Fig. 1b, *psbA*-*trnH* differentiation was markedly inferior to ITS2. The *psbA*-*trnH* barcode cannot distinguish all selected species. Most of them, including *B. pollyphylla*, *B*. *kulingensi*, *B. lineata*, *B*. *sinica*, and *B*. *polyphylla* var. leioclada, cannot be distinguished. The NJ trees according to the *rbcl* sequences were expanded; only *B. discolor* and *B. hirtella* can be distinguished from the others (Fig. 1c). The NJ tree results indicated that *psbA*-*trnH* and *rbcL* were unsuitable for identification of *Berchemia* species. Only the ITS2 region was the most variable, and this sequence was adapted for further Clustal analysis. Both variable sites and deletions in the sequence information can be used to identify species (Jeanmougin et al. 1998).

**Fig. 2:**
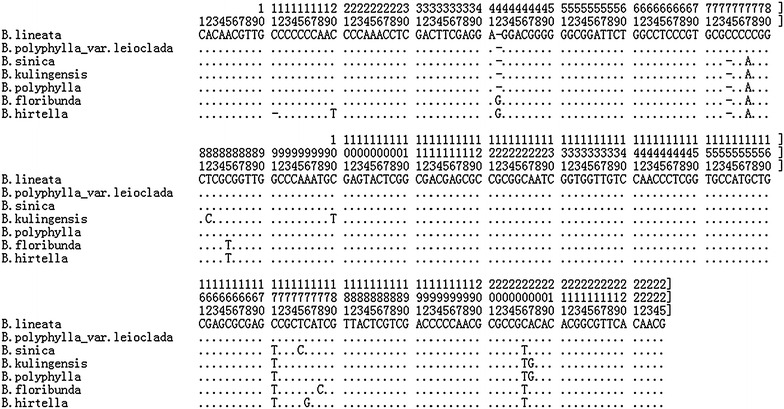
The complete alignment matrix of the *Berchemia* ITS2 sequences

Moreover, the variable sites played an important role in the identification of *Berchemia*. In this study, a deletion was found at site 44 in *B. lineata*, *B. sinica*, *B*. *kulingensis*, *B. polyphylla*, *B. lineata*, and *B*. *polyphylla*. Similarly, a deletion was detected at site 78 in *B*. *lineata* and *B*. *polyphylla* var. *leioclada*; *B*. *hirtella* also showed a deletion at site 17 (Fig. 2). Compared with the sequence regions of *B*. *lineata* and *B. polyphylla* var. leioclada, the other species of *Berchemia* showed variable C-A sites at 76 and C-T sites at 171 and 206. Additionally, the *B*. *sinica* sequence showed variable T-C sites at 175, whereas *B*. *kulingensis* showed variable T-C sites at site 82 and C-T sites at 100 and 207. In addition, *B. kulingensis* and *B. polyphylla* showed variable A-G sites at site 207, *B*. *floribunda* showed variable T-C sites at 178, and *B*. *hirtella* showed variable C-G sites at 176 (Fig. 2).

### Barcoding gap

**Fig. 3:**
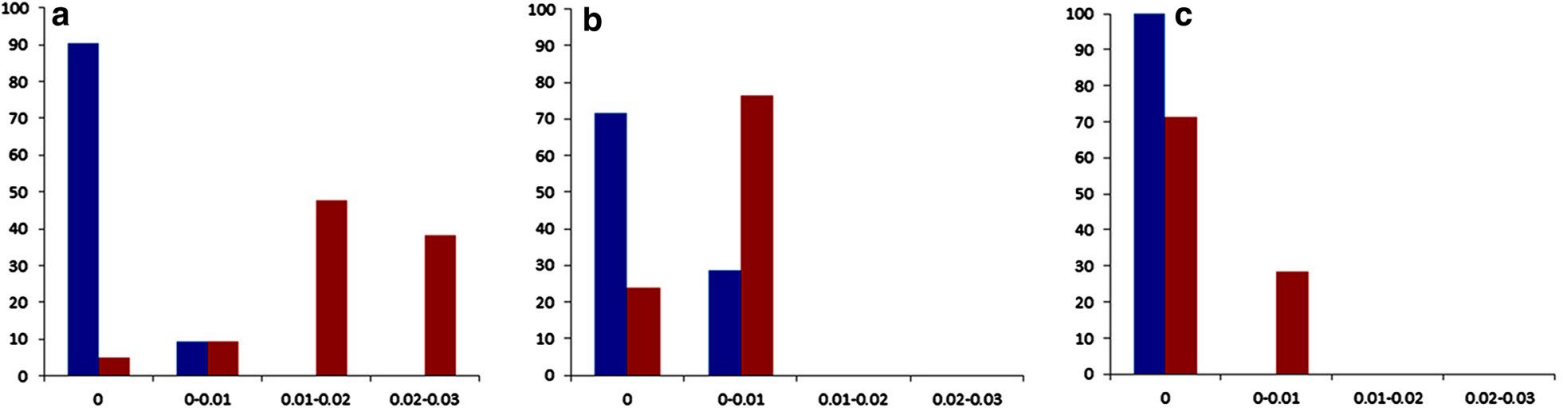
Relative distribution of inter-specific divergence and intra-specific variation of three barcodes **a** ITS2, **b** *psbA*-*trn*H, and **c** *rbcL*, *Blue color* intra-specific, *Red color* inter-specific

To determine whether barcoding gap existed, we assessed the distribution of divergences in *Berchemia* (Fig. 3). The distribution and mean of intraspecific differences were lower than the interspecific divergences, with the highest significance found for ITS2. No obvious barcoding gaps were observed in *psbA*-*trnH* and *rbcL*. Thus, ITS2 can distinguish among *Berchemia* species.

*Berchemia* is a folk tradition medicinal plant with wide geographic distribution in Southwest China. The roots of *B*. *lineata* and other *Berchemia* species have been used as folk medicines to dispel wind and dampness, as well as invigorate blood circulation and analgesia. Additionally, these plants exhibit antitumor, anti-rheumatic, antimicrobial, hepatoprotective, and anti-inflammatory properties (Shen et al. 2010). Currently, *B*. *lineata* and *B*. *polyphylla* var. leioclada are the two primary varieties in herbal medicine markets, and they are traditionally called “Tiebaojin” in specific areas (Yan et al. 2013). However, most of the *Berchemia* species are commonly used as “Tiebaojin” in herbal medicine markets because of their morphological similarity among one another. Furthermore, these plants are typically sold as decoction pieces in the market, which makes morphological analysis difficult to implement once the plants are dried. To ensure food and drug safety, studies have aimed to select a practical and powerful tool to authenticate closely related species. To date, existing methods such as microscopic identification and HPLC fingerprinting cannot effectively distinguish *Berchemia* species (Yan et al. 2013). Therefore, a molecule-based method should be developed.

In this work, DNA barcoding was used to distinguish among eight common and folk medicinal plants of *Berchemia*. The plant *matK* barcode recommended by CBOL was excluded because of its low amplification rate. Thus, we used the ITS2*, psbA*-*trnH*, and *rbcL* regions of nuclear ribosomal DNA to examine a total of 56 samples belonging to eight *Berchemia* species (*B*. *discolor* sequences were obtained from the NCBI nucleotide database). Among the candidate DNA barcodes, the rate of successful identification with ITS2 was 100 % at the species level. Our results highlighted the advantages of using the ITS2 region as a DNA barcode; these advantages include universality, small intraspecific variation but high interspecific divergence, and a small fragment length of approximately 200 bp (Chen et al. 2010). These advantages lead to easy amplification and sequencing (Sun and Chen 2013).

Our study suggested the ITS2 region as the most ideal for *Berchemia* species identification. Pairwise distance analysis validated *Berchemia*, irrespective of the morphological similarities of several subspecies; nevertheless, the analysis failed to validate all the subspecies. Among the varieties *B*. *lineata* and *B*. *polyphylla* var. leioclada, a distance value of zero showed that the ITS2 region cannot differentiate the varieties of these species. Therefore, as indicated by the high degree of sequence variation, the pairwise distance analysis was proven useful in *Berchemia* identification but only up to the species level.

The NJ tree is useful in the identification of most of the species through the formation of monophyletic groups; this tool is also helpful in studying the ancestry and taxonomic positions of some species (Zhou et al. 2008). An issue of concern involves plant taxonomy because *B*. *polyphylla* var. leioclada belongs to the *B*. *polyphylla* subspecies, but it groups with *B*. *lineata*. We assumed that *B*. *polyphylla* may be a variation of *B. lineata* because they demonstrate a very close phylogenetic relationship. This phenomenon indicates that although the two species exhibit a similar morphological appearance, they may not present a close phylogenetic relationship. Hence, species identification at the molecular level is more convenient and efficient.

Clustal analysis is an essential tool used in barcoding (Higgins et al. 1992). In this study, deletion and variable site analysis showed that no barcode was able to differentiate among *Berchemia* spp. at the variation species level, even the ITS2 sequence. With regard to the variation in *Berchemia* spp., in which identification cannot also be achieved via morphological means, other methods can be attempted, such as phytochemical analysis. As previously reported, the quercetin and rutin levels differed between *B. lineata* and *B. polyphylla* var. leioclada. Specifically, *B. lineata* contains more quercetin and less rutin than *B. polyphylla* var. leioclada (Guo et al. 2012).

Ideally, barcodes must exhibit a barcoding gap between interspecific and intraspecific divergences (Meyer and Paulay 2005; Newmaster et al. 2006). To determine the existence of a gap, we assessed the distribution of divergences in classes of 0.001 distance units. The distribution and mean of intraspecific differences were lower than those of interspecific divergences; the highest significance levels were found for ITS2, followed by *psbA*-*trnH* and *rbcL*. The differential efficiency of ITS2 was more effective than that of *psbA*-*trnH* and *rbcL* and more suitable for *Berchemia* spp. in barcode identification. Phylogenetic analysis also showed that *rbcL* and *psbA*-*trnH* were not ideal barcodes in this identification process. The markers mentioned above all belong to the chloroplast genome, hence indicating that these chloroplast genome barcodes may not be suitable for *Berchemia* species identification. Whether this principle can be applied to the identification of other Rhamnaceae plants should be further determined.

## Conclusions

This study demonstrated that DNA barcoding is an effective and useful tool to identify and track various raw materials of *Berchemia* medicinal plants in a cost-effective and efficient manner. This finding also elucidates several taxonomic conflicts among morphologically similar species in the Chinese herb market and provides candidate barcodes for further identification of other Chinese medicinal plants.

## Methods

### Sampling of plant materials

**Fig. 4:**
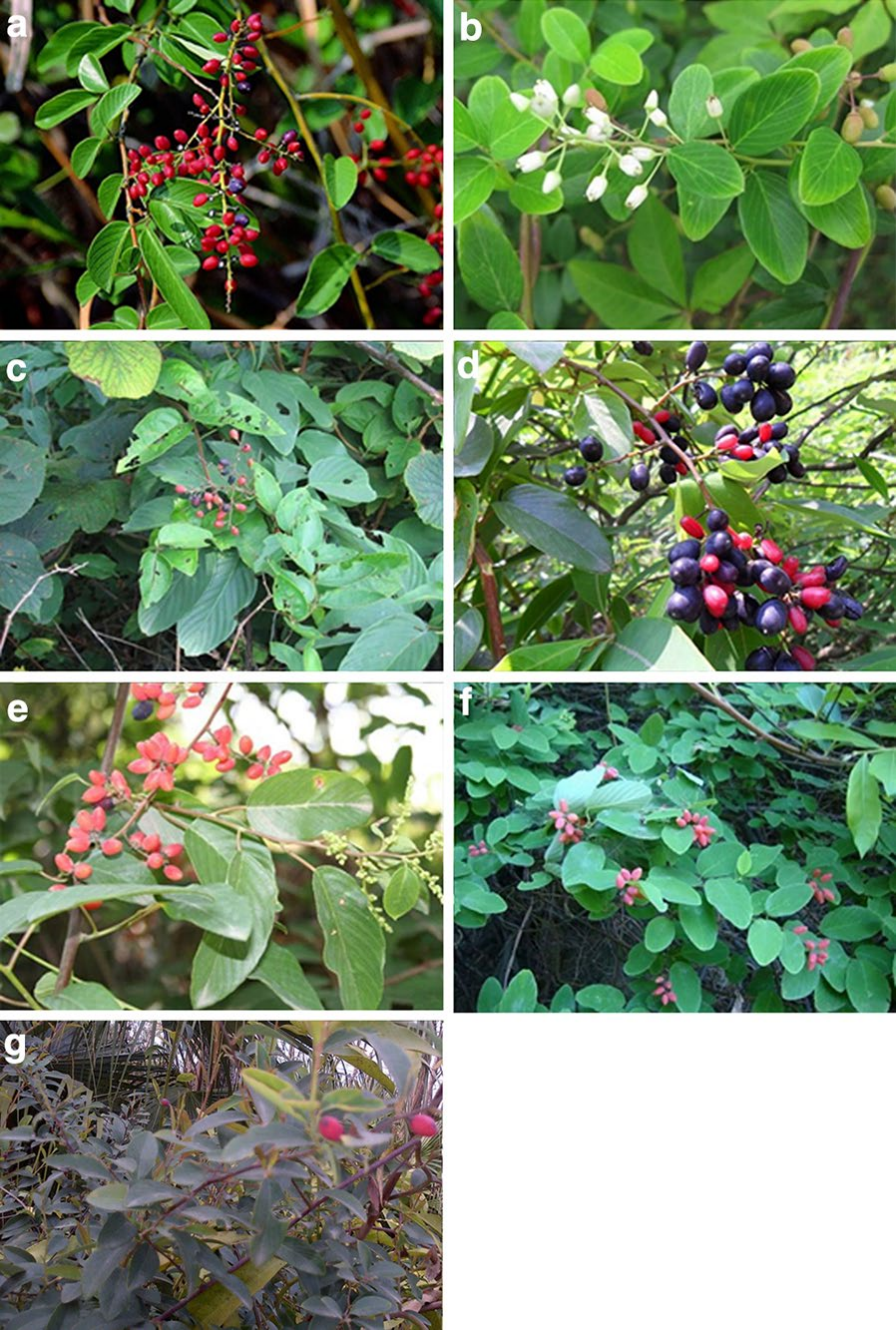
Photos of seven *Berchemia* species. **a** *B. floribunda*, **b** *B. polyphylla*, **c** *B. sinica*, **d** *B. kulingensis*, **e** *B. polyphylla* var. leioclada, **f** *B. lineata*, **g** *B. hirtella*

A total of 55 samples belonging to seven species (Fig. 4), namely, *B. floribunda*, *B*. *polyphylla*, *B*. *sinica*, *B*. *kulingensis*, *B*. *polyphylla* var. leioclada, *B*. *hirella*, and *B*. *lineata*, were sampled from the Guangxi, Guizhou, and Yunnan provinces in China (Table 4). We collected at least three samples for every species. The voucher samples were deposited in the herbarium at the Guangxi Institute of Minority Medicine, Nanning, China. In addition, two *Berchemia* raw material samples were purchased from a local supermarket and pharmacy. Two additional sequences belonging to *B*. *dicolor* were downloaded from the NCBI GenBank and used for comparative studies with the omission of accessions for identical sequence information. All the samples were identified by Liu Shou-yang, a botanist from Guangxi University of Chinese Medicine.

**Table 4:** Specimen voucher, date of collection and Accession numbers of the seven *Berchemia* species

Specimen	Specimen voucher no.	Collection locality	Latitude, and longitude	Date of collection
*Berchemia lineata*	Gmm101	Wuming, Guangxi, China	23.2775, 108.0265	2-Apr-2013
	Gmm102	Nanning, Guangxi, China	22.9704, 108.3643	11-Mar-2013
	Gmm103	Fenghuang, Guangxi, China	23.1483, 108.2550	4-Oct-2013
	Gmm104	Fangcheng, Guangxi, China	21.5296, 108.1716	15-Aug-2013
	Gmm105	Shibing, Guizhou, China	27.1979, 107.9362	10-Set-2013
	Gmm106	Longli, Guizhou, China	26.4236, 106.9669	12-Feb-2013
	Gmm107	Pumiao, Guangxi, China	22.6459, 108.6089	9-Dec-2013
	Gmm108	Tianguan, Guangxi, China	23.8328, 107.3451	25-Mar-2013
	Gmm109	Kunming, Yunnan, China	25.1466, 102.7489	8-May-2013
	Gmm110	Shennongjia, Hubei, China	31.5018, 110.2811	19-Apr-2014
*B. floribunda*	Gmm201	Hezhou, Guangxi, China	24.4493, 111.6069	3-Mar-2013
	Gmm202	Shatian, Guangxi, China	24.3048, 111.4503	11-Apr-2013
	Gmm203	Pingguo, Guangxi, China	23.3318, 107.6717	17-May-2013
	Gmm204	Liuzhou, Guangxi, China	24.3065, 109.0040	18-Nov-2013
	Gmm205	Lianhua, Guangxi, China	24.1594, 110.1244	13-Aug-2013
	Gmm206	Jinxiu, Guangxi, China	24.1228, 110.2234	8-Jun-2013
	Gmm207	Baise, Guangxi, China	23.6189, 106.6550	28-Oct-2013
	Gmm208	Tianguan, Guangxi, China	23.8686, 107.3752	19-Dec-2013
	Gmm209	Kunming, Yunnan, China	21.9219, 101.2792	2-Sep-2013
	Gmm210	Shennongjia, Hubei, China	31.4790, 110.4053	11-Jul-2014
*B. polyphylla*	Gmm301	Mashan, Guangxi, China	23.7286, 108.2027	7-Apr–2013
	Gmm302	Napo, Guangxi, China	23.3947, 105.8710	10-May -2013
	Gmm303	Puding, Guizzhou, China	26.3777, 105.8509	4-Jun-2013
	Gmm304	Wuming, Guangxi, China	23.2866, 108.0720	18-May -2013
	Gmm305	Nanning, Guangxi, China	22.9698, 108.3574	20-Feb-2013
	Gmm306	Tianguan, Guangxi, China	23.8339, 107.2563	27-Apr-2013
	Gmm307	Baise, Guangxi, China	23.7412, 106.4356	18-Nov-2013
	Gmm308	Shennongjia, Hubei, China	31.4569, 110.4266	21-Sep-2013
	Gmm309	Kunming, Yunnan, China	25.1463, 102.7499	9-Mar-2014
	Gmm310	Pingguo, Guangxi, China	23.3537,107.9895	12-Jan-2014
*B. kulingensis*	Gmm401	Shibing, Guizhou, China	27.0124, 108.1447	18-May -2013
	Gmm402	Lushan, Jiangxi, China	29.5808, 115.9856	18-May -2013
	Gmm403	Kunming, Yunnan, China	25.1462, 102.7490	18-May -2013
	Gmm404	Napo, Guangxi, China	23.3984, 105.8189	18-May -2013
	Gmm405	Jingxi, Guangxi, China	23.1634, 106.3467	18-May -2013
*B. sinica*	Gmm501	Kunming, Yunnan, China	25.1460, 102.7492	24-Sep-2013
	Gmm502	Longli, Guizhou, China	26.4224, 106.9686	9-Dec-2013
	Gmm503	Zuoshui, Guizhou, China	33.8007, 108.9149	22-Feb-2014
	Gmm504	Shennongjia, Hubei, China	31.4697, 110.3871	12-May-2104
	Gmm505	Guangzhou, Guangdong, China	23.1840, 113.3672	15-Mar-2014
*B. polyphylla var.* leioclada	Gmm601	Fusui, Guangxi, China	22.6160, 107.9188	6-Mar-2013
	Gmm602	Jingxi, Guangxi, China	23.0306, 106.6587	12-Dec-2013
	Gmm603	Nanning, Guangxi, China	22.9765, 108.3466	9-Mar-2013
	Gmm604	Wuming, Guangxi, China	23.0428, 108.3025	6-Jun-2013
	Gmm605	Huashan, Guangxi, China	23.0338, 108.3008	16-Jun-2013
	Gmm606	Pumiao, Guangxi, China	22.7054, 108.5059	8-Sep-2013
	Gmm607	Basang, Guangxi, China	22.6133, 107.7846	12-Oct-2013
	Gmm608	Shibing, Guizhou, China	27.1034, 108.1265	2-Jul-2013
	Gmm609	Mashan, Guangxi, China	23.6769, 108.2725	4-Aug-2013
	Gmm610	Hezhou, Guangxi, China	24.3172, 111.4549	29-May-2013
*B. hirtella*	Gmm701	Kunming, Yunnan, China	25.1461, 102.7497	13-Jun-2013
	Gmm702	Jingdong, Yunnan, China	24.3963, 100.7885	10-Mar-2013
	Gmm703	Ruili, Yunnan, China	24.0143, 97.8245	8-May-2013
	Gmm704	Zhenkang, Yunnan, China	23.7687, 98.8248	22-Sep-2013
	Gmm705	Menghai, Yunnan, China	21.9820, 100.466	18-Aug-2014

### DNA extraction

Total genomic DNA was extracted from approximately 30–40 mg of dried leaves or 60–70 mg of roots, which were homogenized at 30 Hz with two stainless steel ball bearings in a 2.0 centrifuge tube by using the Plant Genomic DNA Kit (Tiangen Biotech Co., Beijing, China) in accordance with the manufacturer’s protocol. The sample powder was incubated at 65 °C in 750 µL of GP1 buffer. The incubation time was extended from 20 min to 1 h for dried leaves or up to 5 h for roots and rhizomes. The remaining steps followed the manufacturer’s instructions.

### DNA amplification and sequencing

**Fig. 5:**
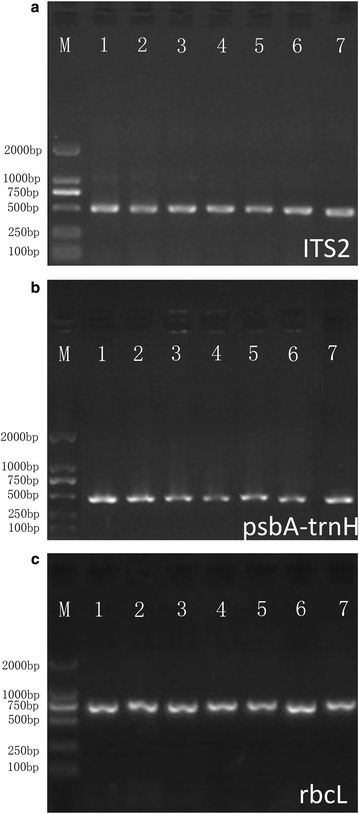
Agarose gel electrophoresis analysis of three PCR products amplified using the primers of ITS2 (**a**), *psbA*-*trnH* (**b**), and *rbcL* (**c**)

PCR was performed using the universal barcode forward and reverse primers for the ITS2, *psbA*-*trnH*, and *rbcL* regions (Table 5) (Kress et al. 2005; Lahaye et al. 2008; Sass et al. 2007; Song et al. 2009). General PCR conditions were adopted, as shown in Table 5 (Chen et al. 2010; Sui et al. 2011). Individual amplifications were performed in 25 µL of a reaction mixture containing 2 × Tag PCR Master Mix (12.5 µL, Aidlab Biotechnologies Co., Beijing, China), 1 µL of each primer (2.5 μmol/L), and double-distilled water (8.5 µL). Approximately 4 µL of PCR products were examined by 1.0 % agarose gel electrophoresis (Fig. 5) and purified using the TIANgel Midi Purification Kit (Tiangen Biotech Co., Beijing, China). The purified PCR products were sequenced using an ABI3730XL sequencer (Applied Biosystems, Foster City, CA) with the amplification primers. All sequence data were submitted to NCBI, and accession numbers were obtained (Table 6).

**Table 5:** List of universal primers and reaction conditions for candidate barcodes

Marker	Name of primers	Primer sequences 5′–3′	PCR conditions	Production expected length (bp)
ITS2	S2F S3R	ATGCGATACTTGGTGTGAAT GACGCTTCTCCAGACTACAAT	94 °C 5 min 94 °C 30 s, 56 °C 30 s, 72 °C 45 s, 40cycles, 72 °C 10 min	491–561
*rbcL*	1f 724r	ATGTCACCACAAACAGAAAC TCGCATGTACCTGCAGTAGC	95 °C 2 min 94 °C 1 min, 55 °C 30 s, 72 °C 1 min, 34 cycles, 72 °C 7 min	729–757
*psbA*-*trnH*	fwd PA rev TH	GTTATGCATGAACGTAATGCTC CGCGCATGGTGGATTCACAATCC	94 °C 5 min 94 °C 1 min, 55 °C 1 min, 72 °C 1.5 min, 30 cycles, 72 °C 7 min	364–470

**Table 6:** NCBI Accession numbers of the ITS2, *psbA*-*trnH* and *rbcL* regions of the obtained six *Berchemia* species and a variety *Berchemia* species

Samples name	Specimen ID	Genbank accession no.		
		ITS*2*	*psbA*-*trnH*	*rbcL*
*B. lineata*	TB01	KP723425	KP723436	KP723396
	TB01-1	KP723426	KP723437	KP723397
	TB01-2	KP723427	KP723438	KP723398
*B. floribunda*	DH02	KP723416	KP723439	KP723399
	DH02-1	KP723417	KP723440	KP723400
	DH02-2	KP723418	KP723441	KP723401
*B. polyphylla*	DY03	KP723433	KP723442	KP723402
	DY03-1	KP723434	KP723443	KP723403
	DY03-2	KP723435	KP723444	KP723404
*B. kulingensis*	KL04	KP723422	KP723445	KP723405
	KL04-1	KP723423	KP723446	KP723406
	KL04-2	KP723424	KP723447	KP723407
*B. sinica*	GE05	KP723419	KP723448	KP723408
	GE05-1	KP723420	KP723449	KP723409
	GE05-2	KP723421	KP723450	KP723410
*B. polyphylla* var. leioclada	GZ06	KP723428	KP723451	KP723411
	GZ06-1	KP723429	KP723452	KP723412
	GZ06-2	KP723430	KP723453	KP723413
*B. hirtella*	GB07	KP723431	KP723454	KP723414
	GB07-1	KP723432	KP723455	KP723415
	GB07-2	HG004838	HG005084	KF181534

### Data analysis

Sequence assembly and generation of consensus sequences were completed using CodonCode Aligner v3.7 (CodonCode Corp., Dedham, MA, USA). The traces were assembled into bidirectional contigs, primer sequences were removed, and all ambiguous base calls were checked manually. Contigs were compared using the MUSCLE multiple sequence alignment algorithms supplemented with the CodonCode Aligner. Genetic variations were analyzed with a Kimura 2-parameter distance matrix, which was constructed using MEGA5.0 software (Ma et al. 2014) and ClustalW (Sun and Chen 2013). A phylogenetic tree was created using the NJ method. Bootstrap test with 1000 replicates was applied to assess the reliability of the phylogenetic trees (Tamura et al. 2011). The inter/intraspecific variations of the samples were calculated as described by Kress et al. (2005) and Song et al. (2009). The obtained sequences were also compared with the existing *Berchemia* species sequences obtained from the NCBI database through BLASTn test (Chen et al. 2010; Ross et al. 2008).
